# Implementation of HPV-based screening in Burkina Faso: lessons learned from the PARACAO hybrid-effectiveness study

**DOI:** 10.1186/s12905-021-01392-4

**Published:** 2021-06-23

**Authors:** Keitly Mensah, Charles Kaboré, Salifou Zeba, Magali Bouchon, Véronique Duchesne, Dolorès Pourette, Pierre DeBeaudrap, Alexandre Dumont

**Affiliations:** 1 Centre Population et Développement (Ceped), Inserm ERL 1244, UMR Institut de recherche pour le développement (IRD) et Université de Paris, 45 rue des Saints-Pères, 75006 Paris, France; 2 Institut de Recherche en Sciences de La Santé (IRSS), Ouagadougou, Burkina Faso; 3 Laboratoire de Recherche Interdisciplinaire en Sciences sociales et Santé (LARISS), Université Ouaga 1, Ouagadougou, Burkina Faso; 4 Pôle Recherche et Apprentissages, Médecins du Monde, Paris, France

**Keywords:** Implementation, Cervical cancer, Process evaluation, Sub-saharan africa, HPV screening, Mixed-method

## Abstract

**Background:**

Cervical cancer screening in sub-Saharan countries relies on primary visual inspection with acetic acid (VIA). Primary human papillomavirus (HPV)-based screening is considered a promising alternative. However, the implementation and real-life effectiveness of this strategy at the primary-care level in limited-resource contexts remain under explored. In Ouagadougou, Burkina Faso, free HPV-based screening was implemented in 2019 in two primary healthcare centers. We carried out a process and effectiveness evaluation of this intervention.

**Methods:**

Effectiveness outcomes and implementation indicators were assessed through a cohort study of screened women, observations in participating centers, individual interviews with women and healthcare providers and monitoring reports. Effectiveness outcomes were screening completeness and women’s satisfaction. Logistic regression models and concurrent qualitative analysis explored how implementation variability, acceptability by women and the context affected effectiveness outcomes.

**Results:**

After a 3-month implementation period, of the 350 women included in the cohort, 94% completed the screening, although only 26% had their screening completed in a single visit as planned in the protocol. The proportion of highly satisfied women was higher after result disclosure (95%) than after sampling (65%). A good understanding of the screening results and recommendations increased screening completeness and women’s satisfaction, while time to result disclosure decreased satisfaction. Adaptations were made to fit healthcare workers’ workload.

**Conclusion:**

Free HPV-based screening was successfully integrated within primary care in Ouagadougou, Burkina Faso, leading to a high level of screening completeness despite the frequent use of multiple visits. Future implementation in primary healthcare centers needs to improve counseling and reduce wait times at the various steps of the screening sequence.

**Supplementary Information:**

The online version contains supplementary material available at 10.1186/s12905-021-01392-4.

## Background

In 2018, cervical cancer (CC) caused 311,000 deaths worldwide, and 90% of these deaths occurred in low- and middle-income countries (LMICs) [1]. The WHO recently called for the elimination of CC as a highly preventable public health problem [2]. CC screening programs worldwide have relied on cytology, visual inspection after coloration with acetic acid (VIA) or human papillomavirus (HPV) detection either alone or combined to screen for cervical precancerous or cancerous lesions at an early stage. Cytological screening was initiated in European countries in the mid-twentieth century and led to a dramatic reduction in the incidence and mortality of CC [3]. Because of its high cost, primary VIA was considered a more cost-effective alternative to be implemented on a large scale in LMICs. However, this strategy showed mitigated success mainly because it requires substantial labor and because its performance can be highly variable [4, 5]. More recently, evidence from two randomized controlled trials in India and South Africa has shown the superiority of primary HPV screening over primary VIA screening to prevent CC occurrence [6, 7], but it is unknown whether primary HPV testing can be successfully replicated in countries characterized by low income, high mortality and weak health systems.

Burkina Faso exemplifies such a situation. Primary VIA screening was implemented at the national level a decade ago, but its effects on reducing CC incidence remain unclear [8]. In this context, the Partnership for Action and Research against Cervical Cancer in West Africa (PARACAO) project launched by the nongovernmental organization (NGO) *Doctors of The World (DOTW)* aims to introduce HPV-based screening at the primary health care level in Ouagadougou, the main city of Burkina Faso. The intervention was based on the premise that primary HPV screening and subsequent management (triage and treatment) should be performed on the same day, the so-called “screen-and-treat” approach, to increase women’s chance of being fully screened and treated [9].

Our primary hypothesis was that the PARACAO could lead to high screening completion among eligible women. The secondary hypothesis was that this new screening is well accepted by women.

The purpose of this study was to simultaneously assess the PARACAO implementation process and its effect on screening completeness and women’s satisfaction to understand the mechanisms underlying the impact of the intervention. We also aimed to examine the effects of contextual factors on implementation and effectiveness outcomes to help implementers design effective implementation strategies.

## Methods

### Context

In 2010, the estimated annual incidence of CC in Burkina Faso reached 1230 women diagnosed and 838 deaths from the disease. As the leading cause of cancer mortality among women in the country [10, 11], the Ministry of Health considered CC a public health priority in 2011, resulting in many actions at the local and national levels. Since April 2016, CC screening has been included as a free service of the national health package for women. In Burkina Faso, the decentralized health system is divided into three levels. The peripheral level operates at the community and district level, providing basic preventive and curative care, it is the entry point in the health system. When necessary, patients can be referred from the primary level to the intermediate or central level, which consists, respectively, of regional hospitals and university or national hospitals [12]. Additionally, biomedical services are divided between public and private (including traditional health practitioners) sectors.

Currently, the national CC control strategy relies on VIA screening and cryotherapy delivered at primary and secondary healthcare facilities nationwide. In addition to VIA screening, colposcopy and more advanced treatment (LEEP, hysterectomy) are available in some private clinics or in the university hospitals of the two main cities (Ouagadougou and Bobo Dioulasso). Despite being covered by the national health plan, women are often required to pay between $1 and 4 US dollars to receive screening because of recurrent shortages in material supply (speculums and gas for cryotherapy) [13].

### Description of PARACAO intervention and its implementation strategy

The PARACAO was developed based on WHO guidelines and on a baseline of formative research [14] (see Additional file 1: Table S1).

The screening strategy included primary HPV testing, triage of HPV-positive women with VIA and prompt treatment of women at need. HPV testing was available either through self-performed or midwife-performed collection of vaginal specimens.

Women with VIA-positive lesions that fulfilled the International Agency for Research on Cancer (IARC) criteria for cryotherapy [14] were supposed to be immediately treated with thermal ablation. Otherwise, they were referred to an identified clinic for the appropriate treatment with the full cost covered by DOTW.

**Fig. 1:**
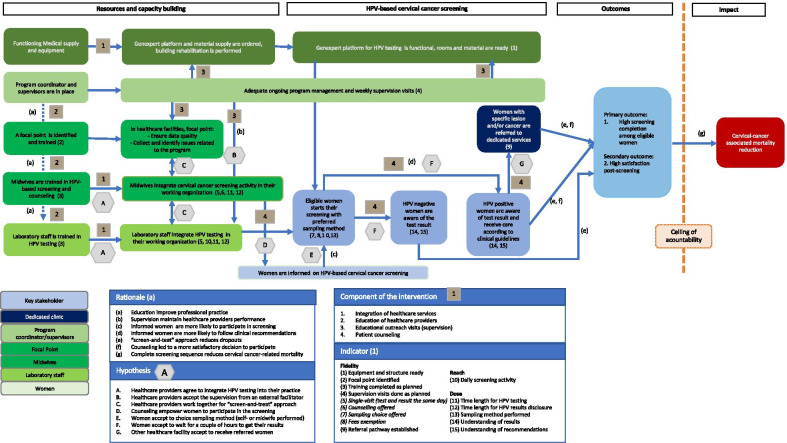
Theory of change (ToC). ToC depicts the various components of the implementation, the assumed pathways through which these components could bring about the targeted changes (mechanisms of change) and the underlying hypotheses that need to hold true for that changes to occur. Indicators are collected at the *facility level (weekly reports and monitoring reports)* and individual level (cohort study)

The entire screening process – from HPV testing to result disclosure, VIA and treatment if needed – was set to be delivered in a single visit, according to the “screen-and-treat” approach (Fig. 1).

The intervention was implemented in 2019 in two urban primary healthcare centers in Ouagadougou. Their general characteristics are shown in Table 1. These sites are believed to be similar to other urban primary health centers in terms of population, activity, staff and equipment. The intervention targeted women attending these health facilities for CC screening or for other healthcare services (family planning, child vaccination, or gynecological issues). Women were considered eligible for HPV-based screening if they were aged 25–55 years old and had no hysterectomy. If they had ongoing menstruation and/or genital infection at the time of the screening, they were considered temporarily ineligible until the condition was resolved. There were no specific geographic criteria for recruitment.

**Table 1:** Healthcare facility characteristics

	Center A	Center B
Physician	1	2
Midwives/nurses	12	9
Birth attendants	11	14
Laboratory staff	4 technical staff, 1 head	2 technical staff, 1 head
Daily prevention consultation	115	90
Daily curative consultation	130	100
Annual target population (25–55 years) for cervical screening	3630	2415

The PARACAO implementation strategy was embedded within the usual model of care delivery of the participating healthcare facilities. Table 2 shows the theoretical framework of the PARACAO. Four components were implemented in each participating facility from May to December 2019 (Fig. 1):

**Table 2:** Components of the PARACAO implementation strategy, underlying theories and assumptions

Component	Description	Theory	Assumption
Integration of healthcare services	Through the process of implementation, healthcare providers and implementers decide on modifications to existing systems, structures, or tasks to offer women the possibility of having an HPV test at the primary healthcare center	Continuum of care for sexual and reproductive health services [11–13]	Integrating HPV testing within primary care enhances both cervical cancer screening and sexual/reproductive health services uptake
Education of healthcare providers	Off-site training of healthcare providers to update their knowledge, persuade them to change their practices, and maintain their competence	Cognitive and learning theories [14]	Education favors the integration of new practices in healthcare settings and improves the quality of cervical cancer screening
Outreach educational visits	A trained supervisor visits each target provider at participating facilities to explore problems, identify possible local solutions, and discuss their concerns	Health promotion, innovation, and social marketing theories [15]	Regular supervisory visits to healthcare providers to help maintain their skills and performance
Patient counseling	Midwives deliver counseling to women at various steps of the screening process: before HPV testing, after the results, after triage and after appropriate treatment if relevant	Women empowerment [16]	Counseling by a trained midwife benefits woman by facilitating a process of informed participation in the context of improved knowledge

*Component 1 – Integration of healthcare services* – The Burkina-based DOTW program coordinator provided changes in structure and equipment required for HPV testing within each participating facility. It consisted of the identification of dedicated rooms for CC screening, building rehabilitation to fit the Genexpert platform requirement (dust, temperature and space) [15] and adequate equipment provision (HPV sampling kits, VIA kits, thermal ablation material, GeneXpert platform, cartridges, furniture, and day-to-day supplies) for the intervention. Tasks for HPV testing were integrated into healthcare providers’ (laboratory staff and midwives) workload to deliver a “screen-and treat” approach.

*Component 2 – Education of healthcare providers* – The DOTW program coordinator organized staff training. Laboratory staff received 5-day off-site training conducted by the CEPHEID training team and a member of the National Tuberculosis Laboratory, who was trained and an expert in the use of GeneXpert. All midwives at participating sites received off-site three-block training. Each training block lasted for 5 days, was conducted by national experts and DOTW national staff and addressed a specific topic: CC screening in general, VIA realization or HPV-based strategy with counseling and sampling options (self or midwife performed).

*Component 3 – Educational outreach visits* – Weekly supervisory visits to each participating facility were intended to ensure that the screening delivery protocol was followed, to assess the fidelity of the project implementation, to identify barriers to implementation and possible strategies to overcome barriers, to reinforce healthcare provider competencies and to verify document and data quality. These visits were performed by DOTW supervisors, and the connection between healthcare providers and supervisors was facilitated by a midwife, identified as a focal point (FP).

In addition, a member of the National Tuberculosis Laboratory, who was trained and an expert in the use of GeneXpert, performed the monthly supervisions of the laboratory staff.

*Component 4 – Patient counseling* – Midwives delivered counseling regarding CC screening to women during their first visit to the participating facility. Part of this counseling consisted of offering the choice between two methods of vaginal sampling for HPV testing: self-performed or midwife-performed collection. Counseling was repeated at each step of the screening process (post-HPV test results, post-VIA and posttreatment) to persuade women to adhere to care management and recommendations. The screening process was entirely free.

All staff (laboratory and midwives) were involved in HPV-based CC screening without receiving financial incentives.

### Study design

PARACAO was the first intervention to deliver HPV-based cervical screening under routine conditions before potential dissemination throughout the country. To better understand the interplay between PARACAO effectiveness and its implementation, we designed a pragmatic hybrid-effectiveness implementation Type III study [16] using mixed methods [17, 18]. Therefore, we focused primarily on PARACAO effectiveness and secondarily on the process evaluation of its implementation. The latter evaluation was performed according to Medical Research Council (MRC) guidelines [19]. Following a baseline of formative research and using consultations with main stakeholders and researchers (see Additional file 1: Table S4), we defined a theory of change [20] (ToC, Fig. 1) that depicts the various components of the implementation strategy, the assumed pathways through which these components could bring the targeted changes (mechanisms of change) and the underlying hypotheses that need to hold true for those changes to occur.

**Fig. 2:**
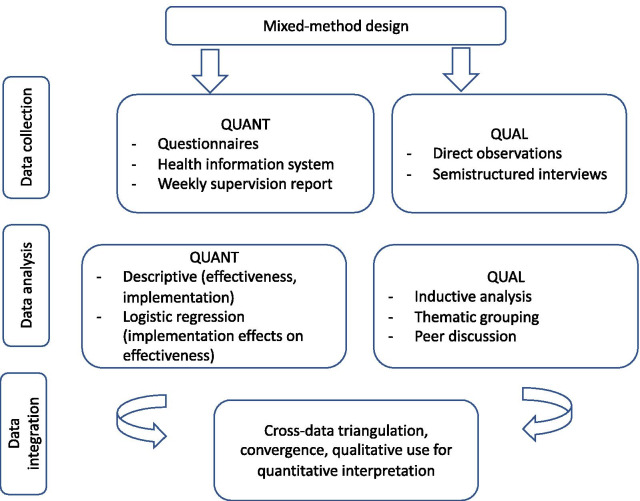
Mixed-method design. Overview of the convergent mixed-method design: data collection, analysis strategy and integration

Based on this ToC, we considered three subcategories of indicators for the process evaluation: fidelity, whether the intervention was delivered as intended; reach, whether women came into contact with the screening offer and appropriate services if needed; and dose, the quantity of intervention implemented. We also assessed whether adaptations of the intervention were required to fit the context of the participating centers and make it more acceptable to women and healthcare providers. The study was conducted from July to December 2019 using a concurrent parallel quantitatively driven mixed-method design [18] (see Fig. 2) that consisted of a cohort study, routine data use, direct observations and semistructured interviews.

### Sampling, data sources and collection

Table 3 outlines the data sources, participants, methods used and outcomes.

**Table 3:** Data sources, participants and outcomes

Method	Participants/recruitment	Sampling	Data collection timing	Outcomes
*QUANTITATIVE DATA*				
Weekly supervision report	Women included in the cohort study, healthcare workers involved in the screening process at participating facilities	Facilities included in the implementation process	January 2018 to December 2019	Implementation outcome Facility-based measure of fidelity
Facility routine health information system	Women included in the cohort study, healthcare workers involved in the screening process at participating facilities	Registries CC screening process (clinical data) HPV testing (laboratory data)	May to December 2019	*Reach*—Number of women screened daily *Dose—*Screening process time, screening steps
Questionnaires (cohort study)	300 women (150/facility) attending facilities for CC screening and eligible for screening as defined by the project	Sample size calculated to provide a 5% accuracy in the measurement of screening completeness Based on an expected screening completeness of 80% and to protect against refusal to participate and dropouts, we decided to include 300 women (n = 150 per facility)	July 1st to October 31st, 2019	*Effectiveness outcomes* Screening completeness Screening process satisfaction: postsampling, postresult and post-VIA if applicable *Implementation outcomes* Individual measure of fidelity Screening steps Context Women’s characteristics
*QUALITATIVE DATA*				
Observations	Women attending facilities for CC screening, healthcare workers involved in the screening process (90 medical visits, 30 laboratory procedures)	Screening activities at facilities Waiting room Screening room Laboratories Performed until saturation is obtained	July 1st to August 31st 2019	CC screening practice Adaptation performed by healthcare workers
Semistructured interviews	20 Women included in the cohort study, 20 healthcare workers involved in the CC screening process	Maximum variation sampling was used to achieve a diverse sample of providers of various qualifications, sexes and seniorities (n = 08 per facility) for individual in-depth interviews. The same method was used to obtain a diverse sample of 20 women in terms of age, religion, ethnicity, and HPV status (n = 10 per facility)	September 1st to November 20th 2019	Women’s CC knowledge, Motivation to undergo screening Experience with HPV-based screening Healthcare workers’ reasons for program adaptation

### Quantitative data

*Cohort study* Women were recruited while attending one of the participating facilities for CC screening and were followed up until completion of the screening sequence. The inclusion and exclusion criteria in the cohort were similar to those considered for screening eligibility.

Data were collected through questionnaires at each step of the screening sequence: after women returned their sample to the laboratory, after they received their HPV test result and after they underwent VIA and treatment if needed. When women did not return to the healthcare center over the 30 days following the sampling, they were contacted by phone.

The questionnaires collected demographic information (age, screening history, living area, and socioeconomic level), CC literacy, intervention delivery data (choice of the sampling method, understanding of test results and recommendations (see Additional file 1: Table S2), date and time for testing, result and postresult management, triage and treatment), and satisfaction at each step of the screening sequence. Socioeconomic (SES) levels were calculated using a wealth index (see Additional file 1: Table S3) according to the asset method [21] and divided into terciles. Participant cervical cancer literacy was assessed after cervical specimen collection using the CC Literacy Assessment Tool (C-CLAT), a 16-item instrument that has been validated in various contexts [22–24]. Each item of the C-CLAT was scored as binary (0 = incorrect, 1 = correct), and the total score computed as the sum of individual items ranged from 0 to 16, with higher scores indicating higher literacy.

*Weekly supervision report* Weekly supervision reports regarding the participating facilities were compiled for the first implementation semester. Weekly supervision reports were retrieved from the project monitoring and evaluation weekly reports starting from the project conception (2018) to the end of the first implementation semester. An in-depth search was made by looking for data related to the implementation process and indicators.

*Facility routine health information system* Each participating facility has a health information system that collects routine data. We retrieved data derived from two registries: CC screening process (clinical data) and HPV testing (laboratory data) for the first implementation semester. Collected clinical data consisted of women’s screening history and description of screening steps (date and time, sampling method, HPV results, VIA results, treatment performed and referral when needed). Laboratory data consisted of day and time for sampling reception, sampling validity, HPV results and genotyping, and time and date for results transferred to midwives..

### Qualitative data

*Direct observations* Direct observations were performed at each participating facility. The anthropologist performed participant observations during the first 2 months of the project implementation. He repeatedly observed various screening activities that took place in the waiting room, in the cervical screening room (sampling performance, the results communication, VIA triage and treatment) and in the laboratory room until saturation was obtained. Realized at various times of the day and of the week over 2 months, the observations covered 90 medical visits and 30 laboratory procedures.

*Semistructured in-depth interviews* Maximum variation sampling was used to achieve a diverse sample of providers of various qualifications, sexes and seniorities (n = 08 per facility) for individual in-depth interviews. The same method was used to obtain a diverse sample of 20 women in terms of age, religion, ethnicity, and HPV status (n = 10 per facility).

The anthropologist conducted semistructured in-depth interviews with the women in their language (Dioula or Mooré) and recorded them between September and December 2019. Women were contacted 2 months after their involvement in the screening process and met outside the facilities. The interviewed women received transportation fee reimbursement as compensation for their time. Semistructured in-depth interviews conducted in French with healthcare providers were performed during their working time and were recorded. All interviews were conducted throughout the implementation stage.

### Outcomes

*Effectiveness outcome* The primary outcome was participant screening completeness. A screening sequence was considered complete in each of the following cases: (1) when an HPV-negative woman was informed of the result of her HPV test, (2) when an HPV-positive woman had a subsequent negative VIA test, or (3) when an HPV-positive woman with a subsequent positive VIA test received appropriate treatment.

The secondary outcome was satisfaction with the screening proposed, measured at each step of the screening sequence – postsampling, postresult and post-VIA if applicable. Assessment was realized through a 3-point Likert scale—*fully agree, agree, disagree*—exploring four dimensions: willingness to repeat the screening; satisfaction regarding the explanation delivered; satisfaction regarding the intervention delivered (sampling performance, VIA and treatment); and willingness to encourage close friends to participate in the same screening procedure.

*Process evaluation* All indicators are listed in Fig. 1.

*Fidelity* The included individual measures of fidelity were derived from the cohort study and were related to the completion of the different steps of the screening and facility-based measures of fidelity that arose from the weekly supervision reports. Fidelity was considered optimal when the elements listed above were successfully completed and the expected rate was 100%.

*Reach* Reach was measured at the facility level and was defined by the daily number of women screened per center. We assumed 20 working days per month with a level of desired achievement set at 4 women screened per day and per center. Data were extracted from the facilities’ routine health information systems.

*Dose* Indicators of dose were assessed at the individual level using data from the cohort study.

### Qualitative data

*Women’s experience* We explored women’s knowledge on CC, their motivation to undergo screening and their experience with HPV-based screening.

*Healthcare workers’ experience* We explored healthcare workers’ experience with the implementation, their relationships with implementers, adaptations made throughout the first stage of implementation and the reasons for deviation from the intervention protocol.

### Data analysis

Quantitative and qualitative analyses were performed independently, and the results were triangulated to look for similarities and discordances. Quantitative data were analyzed using R software version 3.6.3, and qualitative data were analyzed using NVivo software version 12.6.0.

### Quantitative data analysis

*Screening effectiveness and implementation* Data were described using counts and proportions for categorical data, means and standard deviations or medians and interquartile ranges (IQRs) for continuous data. The primary outcome was measured as the proportion of women with a complete screening sequence among screened women. The secondary outcome was measured as the proportion of women highly satisfied, i.e., answering “fully agree” to all of the satisfaction dimensions.

*Effects of implementation variability on effectiveness outcomes* We performed three multivariate logistic regression models to test the association between effectiveness outcomes (screening completeness, postsampling and postresult satisfaction) and individual measures of fidelity and dose. As screening completeness was by definition achieved when a single visit occurred, we performed a subgroup analysis focusing on women who had a multiple-visit approach. Implementation indicators that were considered nondiscriminant (i.e., variables with more than 95% or less than 5% frequency) were not included in the models. When collinearity among variables was detected, only one indicator was selected. The variables were eventually included in the multivariate logistic regression model if they were significantly associated with the outcome in bivariate analysis using a cutoff point of p < 0.20. Each model was adjusted for the health center and demographic information (age, screening history, socioeconomic level, travel cost and literacy score). The associations between post-VIA satisfaction or posttreatement satisfaction and implementation variables were not explored due to the limited group sizes (n = 55 and n = 5).

Comparison of implementation variables and effectiveness outcomes between centers was performed using the chi-square test for binary variables and Student’s t-test or the mood test for continuous variables. The association between demographics and effectiveness outcomes was also analyzed using multivariate logistic regression adjusted for the health center.

*Qualitative data analysis* Direct observation notes were transcribed and compiled through an observation report. All recorded interviews were transcribed and, if necessary, translated in French by the initial interviewer. Reports, transcribed interviews and administrative reports were imported into NVivo software. An inductive analysis of observations and each interview was performed that led to major theme extraction as defined in the thematic analysis approach [25]. After being grouped into a scheme reflecting the implementation process, findings from observations and interviews were triangulated with relevant literature and discussed with a panel of experienced anthropologists.

### Ethical considerations

The study received full ethical approval from the Ethics Committee of Health Research of Burkina Faso (n° 2019-5-064). All women received an information note and signed a consent form before inclusion in the cohort study. Informed consent was obtained from all participants.

## Results

### Quantitative findings

**Fig. 3:**
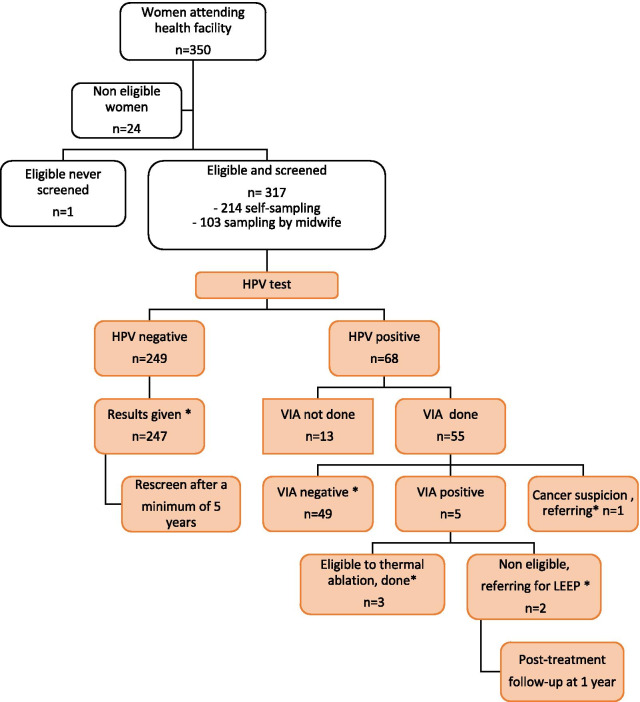
Cohort study data flow. Colored cells indicate WHO guidelines. * indicates the cohort study endpoints

Between July 1st and September 30th, 2019, 350 women attended CC screening services, of which 317 (90%) were eligible for HPV-based screening and eventually included in the cohort study (Fig. 3). Table 4 presents the demographics of the screened women. The majority of them were aged between 25 and 35 years old (61.4%), lived in Ouagadougou (97%) and had no previous screening history (65.3%), with no difference between study sites. Despite the wealthiest women attending Center B and the poorest women attending Center A (p < 0.001), the CC literacy level was similar between centers with a median score of 10/16.

**Table 4:** Participant demographics

	All centers (N = 317)	Center A (N = 160)	Center B (N = 157)	p value
*Age in years (%)*				0.985
25–35 years old	153 (48.3)	78 (48.8)	75 (47.8)	
36–45 years old	142 (44.8)	71 (44.4)	71 (45.2)	
46–55 years old	22 (6.9)	11 (6.9)	11 (7.0)	
*Living area (%)*				0.207
Ouagadougou	295 (97.0)	149 (95.5)	146 (98.6)	
Outside Ouagadougou	9 (3.0)	7 (4.5)	2 (1.4)	
Unknown	14 (4.4)	5 (3.1)	9 (5.7)	
*SES level (%)*				< 0.001
High	111 (35.0)	47 (29.4)	64 (40.8)	
Intermediate	162 (51.1)	79 (49.4)	83 (52.9)	
Low	44 (13.9)	34 (21.2)	10 (6.4)	
*Screening history (%)*				0.115
At least once	97 (30.6)	42 (26.2)	55 (35.0)	
Never	220 (69.4)	118 (73.8)	102 (65)	
*Travel cost for screening process (%)*^a^				0.095
None	59 (18.6)	33 (20.6)	26 (16.6)	
Low	67 (21.1)	38 (23.8)	29 (18.5)	
Intermediate	106 (33.4)	43 (26.9)	63 (40.1)	
High	85 (26.8)	46 (28.7)	39 (24.8)	
*Literacy score (median (IQR))*	10 (3)	10 (2)	11 (3)	0.692

^a^Travel cost is the average amount of money spent by women traveling to healthcare centers during the screening process. It could be none (0$), low (≤ 0.90$), intermediate (≤ 1.80$) or high (> 1.80$). All cost are in US dollars

*Screening completeness* Among the 317 screened women, 299 (94%) had a complete screening sequence (Fig. 3 and Table 5). The more screening steps that were needed, the lower the screening completeness achieved. Of the 68 women (21.4%) who tested HPV positive, 55 (80.8%) had a VIA triage test, and of the 6 HPV + VIA + participants, only 3 were treated. Screening completeness did not differ between centers (Table 5).

**Table 5:** Primary and secondary outcomes

	Overall	Center A	Center B	p value
*Screening completeness*				
HPV-negative women, the results given	247/249 (99.2)	127/128 (99.2)	120/121 (99.2)	1
HPV-positive women, VIA done and negative	49/55 (89.1)	21/25 (84.0)	28/30 (93.3)	0.56
HPV-positive women, VIA positive and treatment provided	3/6 (50.0)	2/4 (50.0)	1/2 (50.0)	1
Women with complete screening sequence^a^	299/317 (94.3)	150/160 (93.8)	149/157 (94.9)	0.84
*Women satisfaction (high vs low)*^*b*^				
Postsampling	205/317 (64.7)	128/160 (80.0)	77/157 (49.0)	< 0.001
Postresults	300/315 (94.6)	146/158 (91.2)	154/157 (98.1)	0.014
Post-VIA	38/55 (69.1)	15/25 (60.0)	23/30 (76.7)	0.29

Data are number of women (%)

^a^Screening sequence was considered complete when an HPV-negative woman was informed of the result of the HPV test, when an HPV-positive woman had a subsequent negative VIA test, or when an HPV-positive woman with a subsequent positive VIA test had an appropriate treatment

^b^Satisfaction was assessed at three steps: after vaginal sampling (postsampling); after women received their test results (postresults); and after the visual inspection if relevant (post-VIA)

*Women’s satisfaction* The proportions of women highly satisfied varied across the screening steps. The highest satisfaction scores were observed after disclosure of the HPV test results (94.6% of women highly satisfied). Postsampling satisfaction was significantly lower in Center B than in Center A, while it was the opposite for post-HPV test result satisfaction (Table 5).

**Fig. 4:**
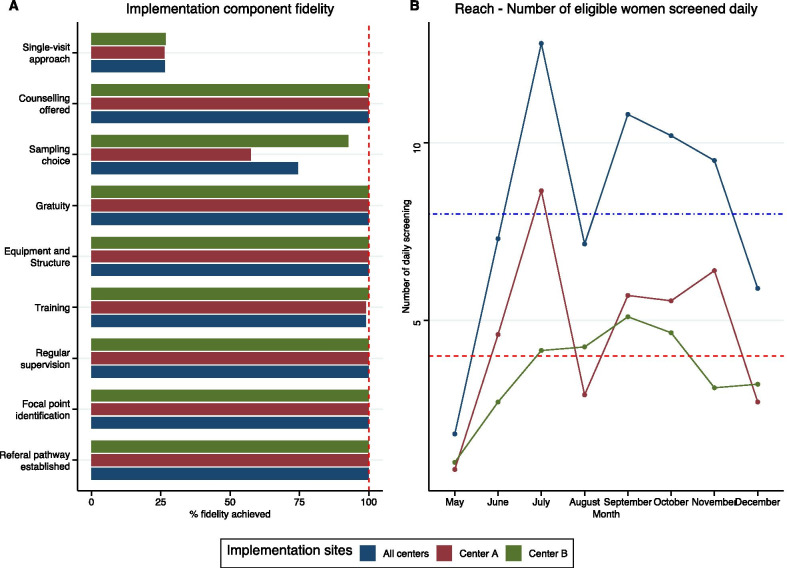
Implementation strategy fidelity and reach. **A** Implementation component fidelity. Level of achievement (%) of the various components of the implementation strategy overall (blue) and by center. The expected level of achievement indicated by the dashed red line. Material-oriented actions received a high level of achievement (equipment and training). **B** Daily screening activity. The reach outcomes are presented as the expected daily number of eligible women screened per center (dashed line) and in all centers (dotted line) according to the initial plan. The overall variations are shown in blue, the variations from Center A are shown in green, and those from Center B are shown in red

*Fidelity* All implementation activities at the facility level in terms of equipment, structure, staff training, supervision and referral system to ensure an optimal environment for HPV-based screening were successfully completed (Fig. 4A). All screened women were counseled by midwives before screening and were exempted from fees (see Additional file 1: Table S5). Midwives offered a choice of sampling method in 72.5% of cases (57.5% in Center A vs 87.9% in Center B, p < 0.001). Only 27.4% of women benefited from a single visit, with no difference between centers (27.5% in center A vs 27.3% in Center B, p = 0.98).

*Reach* In Center A, the trend of daily screened women was uneven, with a peak in July followed by a decrease in August and December (Fig. 4B). In Center B, the screening rate was lower but constantly increased from May to December. In both centers, the reach indicator stabilized at approximately four screened women per day after a 3-month implementation period.

*Dose* The average time to submit samples to a laboratory was 1.22 h (SD = 0.88) and was twice as high in Center B than in Center A (see Additional file 1: Table S6). Overall, the HPV test was performed using self-sampling in 67.5% of cases, with fewer performed in Center B (50%) than in Center A (85%), p < 0.001. The average time between specimen sampling and results disclosure was 2.7 days (SD = 4.01), with no statistically significant difference between centers. A total of 92.4% of women correctly understood their HPV results, and 74.4% of them correctly understood the recommendations made by the midwives for postresult management. The understanding of the results and recommendations was similar in both centers.

*Effect of implementation on effectiveness* Having been screened in a single visit and the time to return samples to the laboratory were not associated with screening completeness (p = 0.43 and p = 0.67, respectively) in the bivariate analysis. In the multivariate analysis (see Additional file 1: Table S7), screening completeness was positively associated with having performed a self-sampling (adjusted odds ratio (ORa) = 4.18; 95% confidence interval (CI) [1.09–17.72]) and having a good understanding of the test results (ORa = 10.62 95% CI [2.10–63.04]). The same factors were found when the analysis was restricted to women who did not benefit from a single visit (see Additional file 1: Table S7), alongside a higher odds of not completing the screening process when the time until result disclosure increased (ORa = 0.16, 95% CI [0.02–0.89]).

Postsampling satisfaction was not associated with any of the dose indicators (see Additional file 1: Table S8). Postresult satisfaction was negatively associated with the level of understanding of the results (ORa = 0.06 95%CI [0.01–0.23]) (see Additional file 1: Table S8). Postresult satisfaction was higher when women received their results within 24–48 h instead of the same day (ORa = 7.03 95% CI [1.40–54.79]).

*Role of demographic characteristics* Demographic characteristics were not associated with screening completeness (see Additional file 1: Table S9). However, postsampling satisfaction decreased among women aged 36–45 compared to that among women aged 25–35 (ORa = 0.52 95% CI [0.30–0.88]). In addition, the adjusted odds of being highly satisfied was almost two times higher among women with a screening history than among women without a screening history (ORa = 1.97 95% CI [1.10–3.63]). Postresult satisfaction was positively associated with the amount of money spent on travel (see Additional file 1: Table S9).

*Effects of context on implementation outcomes* A difference between the reach of the centers was observed at the beginning of the implementation, and these differences can be explained by a “launching effect”. Indeed, the official start of the PARACAO was announced on TV and in newspapers, and despite the announcements indicating that two centers would be involved, the campaign was more focused on one of the two centers.

Likewise, the differences in dose and fidelity found between centers could be explained by differences in internal organization. One center had a dedicated midwife for screening, which resulted in no need for task shifting while she was performing screening. That was not the case in the other center.

In addition, despite having similar populations in their service areas, the two centers had different staffing and activity capacities (Table 2), which could also explain the differences found in effectiveness and implementation outcomes.

### Qualitative findings

*Adaptation* The main adaptation that was implemented to make the PARACAO fit different contexts involves the “screen-and-treat” approach. In one center, HPV test results could be given on the same day as the sampling, but if VIA was needed, the women had to return another day. In the other center, women were systematically asked to return the next day to obtain their test results. In both centers, laboratory staff adapted the initial plan by setting closing times for sample collection that matched their own organizational schedules. The closing times were 10:00 am in one center and 11:00 am in the other, which allowed laboratory staff to perform other routine tests without affecting their working hours (7:00 am to 4:00 pm). Finally, women self-selected themselves and did not come after a certain time of the day, as they knew that screening would no longer be available (see Additional file 1: Table S10).

The counseling was also adapted to improve women’s understanding as perceived by the caregivers. Indeed, health workers expressed concerns regarding the communication of screening results: *“How do we explain HPV-positive results in the local language?”* (Healthcare provider, 12 years of experience). To circumvent these language difficulties, the counseling was often adapted without using either HPV or CC vocabulary: *“It’s ok, you have nothing”* was a sentence commonly used.

*Healthcare worker’s satisfaction* During interviews, the healthcare workers praised the capacity strengthening offered by the training during the implementation process. Indeed, few had received formal training for VIA, and they gained knowledge and self-confidence through the training: *“it helps us to work with more confidence […] now we know the difference when we see the cervix. In terms of knowledge, it really gave us something”* (midwife, 8 years of experience)*.* Likewise, the laboratory staff considered that they were trained for more than HPV testing: *“it’s a plus for us, because you can test many other things than just HPV with this platform. You just have to change the cartridge and the software, and you can test TB, hepatitis B…”* (laboratory staff, 25 years of experience). However, all of them pointed out the additional workload due to the HPV screening strategy as expressed by a midwife “before it was simple, now we have to take time to explain, explain again before doing the test, and it takes much more time” (midwife, 8 years of experience) and suggested some solutions, such as “we said to the NGO that maybe it would be better to group women and screen them once a week […]” (laboratory staff, 7 years of experience).

*Women’s satisfaction* Patient interviews showed that women were motivated by their peers to obtain screening: *“A colleague of mine, she did the test and she told me that I should do it, it’s free”* (woman, 29 years old, high school education). This was particularly the case since the PARACAO was perceived as *“a novelty brought by white people, so it has better quality than the old method (VIA test*)” (woman 34 years old in the waiting room, previously screened, primary school education). However, they often expressed their disappointment about the counseling quality when asked about their screening experience: *“the explanations the midwife gave me were… not good. She didn’t explain to me anything about the cancer before the test. When I came back for the results, she explained, but just a little bit.”* (woman 30 years old, college education, never screened). This disappointment was higher among women who had heard of the screening procedure from peers: *“My cowives told me that they explain the cancer causes and give advice. […] but when I came, nothing like this happened”* (woman 33 years old, never screened, primary school education, right after her screening). Furthermore, the choice offered for sampling collection raised mixed feelings among women and may have affected satisfaction. Some participants believed that the healthcare providers should have decided instead of them, while others were happy with the opportunity offered. From interviews, it appeared that the women with the highest level of education and prior awareness of the screening novelty were more likely to choose self-sampling, as it respected their intimacy: *“I appreciated it [the self-sampling] more, because with the old version and the speculum, it hurts. This time, I didn’t feel anything. And I didn’t have to lay down, it was good.*” (woman, 26 years old, previous screening, college education). Women without prior knowledge and with a lower level of education relied more on caregivers: *“I think that if the midwife does the sampling it’s better […] I trust them more, it’s their job, not mine”* (woman 33 years old, never screened, no education).

## Discussion

**Fig. 5:**
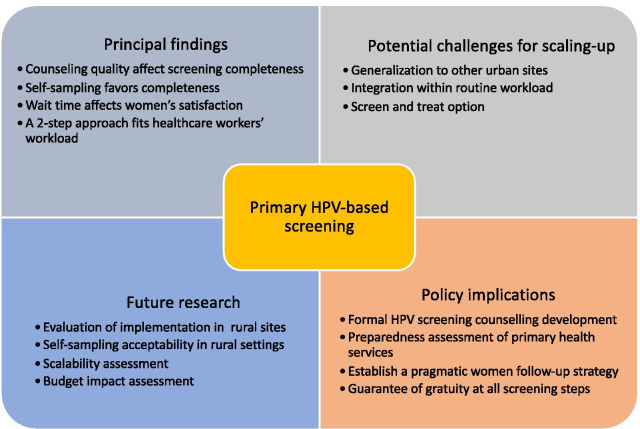
Study findings policy implications

This study provides insight into the implementation process of an HPV-based cervical cancer screening program (Fig. 5), which is important for the future expansion of HPV-based screening of CC as envisioned by the WHO [26]. We found that the healthcare providers of both facilities adhered relatively well to the various components of the screening implementation and accepted integrating HPV screening into their work schedules. However, they had to adapt the strategy by moving from a “screen-and-treat” approach to a multiple-visit approach. This adaptation was the result of a dialogue between midwives and laboratory staff in both centers to facilitate the integration of screening activities into the existing work structure, revealing a context modification according to Stirman’s classification [27]. As observed in other contexts, this type of adaptation to fit an organization could enhance sustainability [28, 29].

Despite the modifications, both facilities nearly achieved the level of desired screening activity (4 women screened per day), and although women rarely received results on the same day that they provided the samples, 94% of them had a complete screening sequence. These findings mitigate the usual paradigm whereby multiple visits result in a high dropout rate among women [30, 31].

Through this analysis, we identified aspects of the implementation process critical to screening completeness and satisfaction. The main determinant for screening completeness was the understanding of the results by the women. This result is linked to the poor counseling quality as expressed by the women. The importance of counseling content on screening uptake, treatment adherence and health behavior has been demonstrated for other health conditions [32, 33], and evidence indicates that it is more important than counseling duration [34]. In our study, midwives complained about the lack of adequate words in the local language for delivering the correct message. This issue could be resolved by adapting messages to the local language with the help of social scientists or linguists. Indeed, the literature shows that effective health communication requires both cultural and language adaptations [35, 36]. Self-sampling, whether chosen or not, was also found to be associated with screening completeness. This method prevents women from undergoing a gynecological examination during the first screening step and thus diminishes the embarrassment associated with this examination, which is one of the main barriers to cervical screening [37–39]. In addition, self-sampling as an empowering tool [40] may have played a role in this high completion rate. Other hypotheses could be raised to explain the high completion rate. First, many women were interested in this new screening strategy, as it was imported by westerners, which was perceived as a “guarantee of quality”, leading to reduced dropouts due low-quality health services [41, 42]. A similar situation was found by Doctors Without Borders in Niger, where NGOs targeting health issues were positively perceived by the population [43]. DOTW is an NGO known for its work with communities, which may explain why women were more prone to participate and return to the health center when contacted by DOTW [44].

Moreover, the program was implemented in an urban area within a major city, where constraints associated with healthcare access—road conditions, field work, distance—are less important [38, 45], which could also explain this high completion rate [37].

Of note, we found that women living farther from the participating facilities were more likely to be satisfied. This counter-intuitive result could be explained by the fact that these women were attracted by the novelty of the screening and were unfamiliar with the center, and hence had different expectations from those living in the immediate vicinity of the center.

We found that screening completeness and patient satisfaction were higher for a rapid two-step approach (within 48 h) than for a single-visit approach. This suggests that cumulative wait time at the healthcare center may hinder screening satisfaction and that the two-step strategy might better fit in this context. Observations and interviews in healthcare centers revealed that wait times were related to health service organization and staffing. This raises questions about the potential effect of adding new services or, more generally, integrating new services into saturated centers. Studies report conflicting results, with some showing that integration may improve service use without changing health outcomes due to imposing additional workloads [46] and others showing that integration has positive long-term effects [47]. Although further research is needed to clarify the effect of CC screening integration [48], our findings highlight the implementation components that are essential to providing HPV-based screening at the primary-care level [49]. If the single-visit screen-and-treat approach is to be prioritized in a future national strategy, important changes to staffing or in work structure will be required. Either more lab technicians will be needed, which seems quite unlikely with the current limited health budget; midwives will need to be allowed to perform HPV testing, which could facilitate a one-step approach [50]; or reliable point-of-care HPV testing that does not require a traditional laboratory setting must be developed [51]. Such alternatives need to be further explored.

Some limitations to our results are worth noting. First, the assessment encapsulated the early stages of implementation and may not capture all aspects of the project in terms of reach, dose and fidelity. However, we believe that early adaptations are of central importance and will shape the final form of the project. Furthermore,, observations performed during this first stage may have modified healthcare workers’ behavior (Hawthorne effect). However, we tried to mitigate this effect through the observation process itself (establishing rapport, long-time observation) and later through a triangulation between all qualitative and quantitative data. Similarly, we may have encountered a recall bias regarding women’s screening experience as interviews were performed 2 months after the screening started. This could have led to overestimation of extreme experiences (positive or negative). However, findings from interviews were convergent with findings from the cohort study, which suggests that this bias may have been reduced. Our study was limited to two centers, which makes generalization of our findings difficult. We tried to overcome this issue through our cohort study sample size and maximum variation during interviews with women to reflect various screening situations. In addition, the study sites were located in urban areas without access restrictions. Maintaining a single visit for the screen-and-treat approach may turn out to be more important in such settings, but more research is necessary to confirm this. Another limitation that should be acknowledged is the absence of information on the outcomes of the women who were referred for cancer or large treatment management.

*Policy implications* Our results highlight that further research is needed to grasp the potential success of HPV implementation in rural settings (Fig. 5). However, we have demonstrated that some gaps could be filled in the near future to drive HPV-based screening in urban settings. First, offering formal systematized counseling at each step would improve women’s understanding while reducing healthcare workers’ wording issues. Then, an individual facility preparedness assessment needs to be performed before implementation to account for the existing workload and healthcare workers’ needs in terms of adaptation. Additionally, as a multiple-visit approach will probably be used, establishing a pragmatic follow-up strategy through community health workers and accurate registries would help in reducing this risk. Finally, a guarantee for free-of-charge CC screening is one of the most important steps to promote successful implementation.

## Conclusion

Despite some limitations, we believe that our results have important implications for future programs and healthcare providers in low-resource settings, especially in the context of expanding HPV-based screening strategies at the primary-care level.

First, the single-visit approach should not be the ultimate goal for HPV-based cervical screening, and the multiple-visit approach is an acceptable option in the urban context as long as results are given within 48 h and adequate counseling is provided. Baseline assessment is needed to adapt the intervention to workload and staffing constraints to reduce the wait times for testing and result disclosure as much as possible. Efforts should be made to involve patients in deciding their sampling collection method and to counsel them with appropriate language and wording. These results will help decision-makers design effective future HPV-based screening implementations in resource-constrained settings.

## Supplementary Information

**Additional file 1.** Supplementary material relative to methods applied and results presented in the main manuscript

## Data Availability

The datasets used and/or analyzed during the current study are available from the corresponding author upon reasonable request.

## Abbreviations

CC: Cervical cancer
CI: Confidence interval
DOTW: Doctors of the World
FP: Focal point
HPV: Human papilloma virus
IARC: International Agency for Research on Cancer
LEEP: Loop electrosurgical excision
LMICs: Low- and middle-income countries
MRC: Medical Research Council
NGO: Nongovernmental organization
ORa: Adjusted odds ratio
PARACAO: Partnership for Action and Research against Cervical Cancer in West Africa
VIA: Visual inspection with acetic acid
WHO: World Health Organization
