# Supplementary material for: Implementation of HPV-based screening in Burkina Faso: lessons learned from the PARACAO hybrid-effectiveness study
Source: BMC Womens Health. 2021 Jun 23;21:251. doi: 10.1186/s12905-021-01392-4 (PMC8220722; doi:10.1186/s12905-021-01392-4)
Supplement: Supplementary file 1 — Additional file 1. Supplementary material relative to methods applied and results presented in the main manuscript [file 12905_2021_1392_MOESM1_ESM.pdf]

SUPPLEMENTARY MATERIAL

Implementation of HPV-based screening in Burkina Faso: lessons learned from the PARACAO hybrid-effectiveness study.

Keitly Mensah[1], Charles Kaboré[2], Salifou Zeba[3], Magali Bouchon[4], Véronique Duchesne[1], Dolorès Pourette[1], Pierre DeBeaudrap[1], Alexandre Dumont[1]

1. Centre Population et Développement (Ceped), Institut de recherche pour le développement (IRD) et Université de Paris, Inserm ERL 1244, Paris, France.
2. Institut de recherche en sciences de la santé (IRSS), Ouagadougou, Burkina Faso
3. Laboratoire de Recherche Interdisciplinaire en Sciences sociales et Santé (LARISS), Université Ouaga 1, Ouagadougou, Burkina Faso
4. Pôle Recherche et apprentissages, Médecins du Monde, Paris, France

Corresponding author :

Keitly.mensah@ird.fr

Centre Population et Développement (Ceped)

45 rue des Saints-Pères

75006 Paris

I - Method

1. Formative research results

Formative research has shown the need to improve cervical screening uptake and implementation components. These issues were discussed through multiple meetings until a consensus was reached regarding a final theory of change and the related implementation components.

Table S1 – Needs and implementation components aiming to address them	
**Needs**	Implementation component
**Increase cervical cancer screening awareness and improve knowledge among women**	Counseling at each screening step – before and after sampling, after results, after VIA and after treatment
**Empower women**	Offer a choice of sampling method
**Increase acceptance among women**	Offering a self-sampling method; fee exemption
**Regular VIA training to improve VIA quality**	Midwife training and regular supervision
**Knowledge of HPV testing**	Laboratory staff training and supervision
**Avoid material shortages**	Provide equipment and material + establish a dedicated fund for the healthcare centers
**Integration of cervical screening within routine practices**	Integration of cervical cancer screening in maternity services
**Monitoring data on screening uptake**	Establish training and material to monitor monthly screening data
**Avoid multiple visits to health facilities for screening**	Providing a single-visit approach with sampling, testing, results, and treatment provided in a single day

2. Assessment of women's understanding of the results and recommendations

The table below presents the assessment of the understanding and misunderstanding of results and recommendations. The understanding of test results and the understanding of recommendations related to care management and treatment were assessed after the disclosure of the results and after VIA/treatment if relevant.

Table S2 – Assessing understanding			
		**Laboratory results**	
		**HPV -**	**HPV +**
**Understanding of results**	Yes	Woman says the result is normal	Woman says the result is abnormal
	No	Woman says the result is abnormal	Woman says the result is normal
**Understanding of recommendations**	Yes	Woman says that she has to come back in 5 years	Woman says she needs more exploration and/or a treatment and/or to see another doctor
	No	Woman says that she needs more exploration and/or a treatment	Woman says she needs to come back in 5 years

3. Wealth Index Calculation

We used the World Food Program (WFP) tutorial to calculate the wealth index.

From our survey, we selected the variable of interest. Through the frequency table, we suppressed the variables that occurred at a greater than 95% frequency or less than 5% frequency, as they would not be relevant.

The final variables were recoded as binary variables. If a variable had more than 2 levels, using the literature and field knowledge, we recoded them to reflect poorer and wealthier households.

Table S3 – Wealth index asset coding

**Name of variable**	Poorer	Wealthier
Marital status	0 = single or widow or divorced	1 = married
Education level	0 = lower than middle school	1 = middle school or higher
Partner education level	0 = lower than middle school	1 = middle school or higher
Principal means source	0 = spouse or family	1 = self
Ability to save money	0 = no	1= yes
Water access	0 = forage or stocked	1= tap water
Family size	0 = over 7 people	1= 7 or less
Travel access	0 = foot or bicycle	1= car or motorbike or taxi
Employment status	0 = student or unemployed	1= housewife
Type of work	0 = student, informal sector	1= private sector, business woman

Once all the variables were recoded, we conducted a principal component analysis (PCA) to create the wealth index. In the case of missing data, we imputed data using the mean method. Here, the first PCA component explains 41% of the total variance and is used to create the wealth index.

We then extracted the scores for each variable and applied them to the variables included in the composite index. The results were separated by tercile, and we used this wealth level for the analysis.

4. Process evaluation

The table below presents the participants in the meeting to create an implementation strategy.

Table S4 – Status and role of participants involved in implementation strategy development

**Status**	Role (nb)
**Local implementation team (based in Ouagadougou)**	Project leader (1)<br>Medical coordinator (1)<br>Logistical head (1)<br>Communication head (1)
**Headquarter implementation team (based in Paris)**	Social science head (1)<br>Research director (1)<br>Project manager (2)<br>Medical coordinator (1)<br>Volunteers (2)
**Research team**	Epidemiologist (2)<br>Social scientist (2)<br>PhD student (1)
**Local policy makers**	Head of Medical Districts (2)<br>Ministry of Health<br>Traditional community leaders
**Local caregivers**	Head of health centers (7)<br>Head of laboratories (7)

II- Results

1. Implementation fidelity at the individual level

Table S5 – Individual level fidelity achievement				
	**Overall (N=317)**	**Center A (N=160)**	**Center B (N=157)**	P value
Single visit				0.982
**Yes**	87 (27.4)	44 (27.5)	43 (27.4)	
**No**	230 (72.6)	116 (72.5)	114 (72.6)	
Choice of sampling method (n,%)				< 0.001
**Yes**	230 (72.6)	92 (57.5)	138 (87.9)	
**No**	87 (27.4)	68 (42.5)	19 (12.1)	
Counseling offered (n,%)				1
**Yes**	317 (100)	160 (100)	157 (100)	
**No**	0 (0)	0 (0)	0 (0)	
Gratuity (n,%)				1
**Yes**	317 (100)	160 (100)	157 (100)	
**No**	0 (0)	0 (0)	0 (0)	

2. Implementation at the individual level

The table below shows the intensity of implementation overall and within each center.

**Table S6 – Implementation at the individual level**				
	**Overall (N=317)**	**Center A (N=160)**	**Center B (N=157)**	P value
**Sampling method (n,%)**				< 0.001
Performed by midwives	103 (32.5)	24 (15.0)	79 (50.3)	
Self-performed	214 (67.5)	136 (85.0)	78 (49.7)	
**Wait time for submitting a sample to a laboratory (mean (SD))**	1.22 (0.88)	0.74 (0.71)	1.69 (0.76)	< 0.001
**Time to result disclosure (mean, SD)**	2.737 (3.92)	2.82 (3.70)	2.65 (4.14)	0.695
**Time to result disclosure (n,%)**				0.287
Same day	87 (27.4)	44 (27.5)	43 (27.4)	
24-48 h postsampling	113 (35.6)	51 (31.9)	62 (39.5)	
>48 h postsampling	117 (36.9)	65 (40.6)	52 (33.1)	
**Understanding of results (n,%)**				0.707
Yes	293 (92.4)	147 (91.9)	146 (93)	
No	24 (7.6)	13 (8.1)	11 (7.0)	
**Understanding of recommendations (n,%)**				0.628

Yes	236 (74.4)	124 (77.6)	115 (73.2)
No	81 (25.6)	39 (24.4)	42 (26.8)

3. How implementation variability affected outcomes

a. Association between implementation and screening completeness

Table S7 – Logistic regression exploring implementation factors associated with screening completeness

		All women screened (N=317)				Women with no single visit (N=230)			
		Incomplete screening	Complete screening	Unadjusted OR	Adjusted OR* (CI 95%, p)	Incomplete screening	Complete screening	Unadjusted OR	Adjusted OR* (CI 95%, p)
**Fidelity**									
Choice of sampling method	No	8 (9.2)	79 (90.8)			7 (11.3)	55 (88.7)	-	-
	Yes	10 (4.3)	220 (95.7)	2.23 (0.82-5.85, p=0.104)	1.78 (0.45-6.80, p=0.400)	8 (4.8)	160 (95.2)	2.55 (0.86-7.41, p=0.084)	2.59 (0.49-14.02, p=0.255)
Single visit	No	15 (83.3)	215 (71.9)	-	-	-	-	-	-
	Yes	3 (16,7)	84 (28.1)	1.95 (0.62-8.59, p=0.300)	-	-	-	-	-
**Dose**									
Sampling method	Performed by midwives	9 (8.7)	94 (91.3)	-	-	9 (11.5)	69 (88.5)	-	-
	Self-performed	9 (4.2)	205 (95.8)	2.18 (0.83-5.76, p=0.110)	4.18 (1.09-17.72, p=0.041)	6 (3.9)	146 (96.1)	3.17 (1.10-9.80, p=0.035)	8.48 (1.66-61.48, p=0.017)
Waiting time for submitting a sampling to a laboratory	Mean (SD)	1.2 (0.9)	1.3 (1.0)	1.12 (0.68-1.95, p=0.673)	-	1.1 (0.8)	1.3 (1.0)	1.33 (0.76-2.60, p=0.353)	-
Understanding of results	No	9 (37.5)	15 (62.5)	-	-	9 (42.9)	12 (57.1)	-	-
	Yes	9 (3.1)	284 (96.9)	18.93 (6.54-55.82, p<0.001)	10.62 (2.10-63.04, p=0.006)	6 (2.9)	203 (97.1)	25.37 (7.93-87.83, p<0.001)	8.76 (1.31-96.99, p=0.041)
Understanding of recommendations	No	12 (14.8)	69 (85.2)	-	-	11 (22.4)	38 (77.6)		
	Yes	6 (2.5)	230 (97.5)	6.67 (2.49-19.76, p<0.001)	4.89 (0.91-27.04, p=0.060)	4 (2.2)	177 (97.8)	12.81 (4.14-48.22, p<0.001)	8.75 (0.79-93.90, p=0.063)

Time to result disclosure	Same day	3 (3.4)	84 (96.6)	-	-		-	-	-
	24-48 h	3 (2.7)	10 (97.3)	0.76 (0.14-4.22, p=0.745)	0.70 (0.09-4.90, p=0.714)	3 (2.7)	110 (97.3)		
	>48 h	12 (10.3)	105 (89.7)	3.20 (0.98-14.38, p=0.079)	2.68 (0.61-15.20, p=0.218)	12 (10.3)	105 (89.7)	0.24 (0.05-0.78, p=0.030)	0.16 (0.02-0.89, p=0.055)
Healthcare facility	Center A	150 (93.8)	10 (6.2)			8 (6.9)	108 (93.1)		
	Center B	149 (94.9)	8 (5.1)	1.24 (0.48-3.34, p=0.658)	1.22 (0.29-5.22, p=0.785)	7 (6.1)	107 (93.9)	1.13 (0.39-3.33, p=0.816)	1.04 (0.17-6.81, p=0.967)

* Odds ratio adjusted for fidelity variables, dose variables, primary healthcare center, woman’s age, screening history, socioeconomic level, travel cost and literacy score

b. <u>Association between implementation and women's satisfaction</u>

The regression model exploring the association between postresult satisfaction and implementation variables includes significant variables associated with postsampling satisfaction.

Table S8- Logistic regression exploring implementation factors associated with screening satisfaction					
		Unsatisfied	Highly satisfied	Unadjusted OR	Adjusted OR* (CI 95%, p)
Postsampling satisfaction					
**Fidelity**					
Choice of sampling method	No	23 (26.4)	64 (73.6)		
	Yes	89 (38.7)	141 (61.3)	0.57 (0.325, 0.97, p=0.043)	0.92 (0.48-1.75, p=0.796)
**Dose**					
Waiting time for submitting a sample to a laboratory	Mean (SD)	1.4 (0.8)	1.3 (1.0)	0.90 (0.71-1.15, p=0.408)	-
Postresult satisfaction					
**Fidelity**					
Single visit	No	11 (4.8)	219 (95.2)		
	Yes	6 (6.9)	81 (93.1)	0.68 (0.25-2.02, p=0.458)	-
**Dose**					
Understanding of results	No	7 (29.2)	17 (70.8)		
	Yes	10 (3.4)	283 (96.6)	0.09 (0.03-0.26, p<0.001)	0.06 (0.01-0.23, p<0.001)
Time to result disclosure	Same day	6 (6.9)	81 (93.1)	-	-
	24-48 h	2 (1.8)	111 (98.2)	4.11 (0.92-28.55, p=0.088)	7.03 (1.40-54.79, p=0.030)
	>48 h	9 (7.7)	108 (92.3)	0.89 (0.29-2.56, p=0.830)	2.84 (0.72-12.17, p=0.141)
Healthcare facility	Center A	11 (34.4)	21 (65.6)	-	-
	Center B	1 (2.8)	35 (97.2)	18.33 (3.22-347.31, p=0.007)	36767.48 (61.77-351280352060.21, p=0.032)

* Odds ratio adjusted for fidelity variables, dose variables, primary healthcare center, woman's age, screening history, socioeconomic level, travel cost and literacy score

4. Role of women's characteristics in screening outcomes

Table S9 – Logistic regression between women's characteristics and screening outcomes

		Screening completeness				Postsampling satisfaction				Postresult satisfaction			
		**Complete**	**Incomplete**	**Unadjusted OR**	**Adjusted OR**	**Unsatisfied**	**Satisfied**	**Unadjusted OR**	**Adjusted OR**	**Unsatisfied**	**Satisfied**	**Unadjusted OR**	**Adjusted OR**
**Age in years**	25-35	146 (95.4)	7 (4.6)			46 (30.1)	107 (69.9)			8 (5.2)	145 (94.8)		
	36-45	134 (94.4)	8 (5.6)	1.25 (0.44-3.64)	1.33 (0.45-3.98)	62 (43.7)	80 (56.3)	0.55 (0.34-0.89)	0.52 (0.30-0.88)	8 (5.6)	134 (94.4)	0.92 (0.33-2.58)	0.80 (0.27-2.36)
	46-55	19 (86.4)	3 (13.6)	3.29 (0.67-12.99)	2.92 (0.57-12.18)	4 (18.2)	18 (81.8)	1.93 (0.68-6.97)	2.09 (0.66-8.17)	1 (4.5)	21 (95.5)	1.16 (0.20-22.04)	1.31 (0.19-26.94)
**SES level**	High	105 (94.6)	6 (5.4)			33 (29.7)	78 (70.3)			6 (5.4)	105 (94.6)		
	Intermed.	152 (93.8)	10 (6.2)	1.15 (0.41-3.47)	1.08 (0.36-3.47)	67 (41.4)	95 (58.6)	0.60 (0.36-1.00)	0.59 (0.33-1.05)	7 (4.3)	155 (95.7)	1.27 (0.40-3.91)	2.00 (0.57-6.97)
	Low	42 (95.5)	2 (4.5)	0.83 (0.12-3.78)	0.48 (0.06-2.69)	12 (27.3)	32 (72.7)	1.13 (0.53-2.52)	1.06 (0.42-2.77)	4 (9.1)	40 (90.9)	0.57 (0.16-2.33)	2.03 (0.45-10.04)
**Screening history**	Never	210 (95.5)	10 (4.5)			85 (38.6)	135 (61.4)			10 (4.5)	210 (95.5)		
	At least once	89 (91.8)	8 (8.2)	1.89 (0.70-4.94)	2.02 (0.71-5.60)	27 (27.8)	70 (72.2)	1.63 (0.98-2.78)	1.97 (1.10-3.63)	7 (7.2)	90 (92.8)	0.61 (0.23-1.73)	0.48 (0.17-1.42)
**Travel cost**	None	55 (93.2)	4 (6.8)			23 (39.0)	36 (61.0)			6 (10.2)	53 (89.8)		
	Low	61 (91.0)	6 (9.0)	1.35 (0.37-5.52)	1.32 (0.34-5.52)	26 (38.8)	41 (61.2)	1.01 (0.49-2.07)	1.00 (0.45-2.21)	6 (9.0)	61 (91.0)	1.15 (0.34-3.89)	1.26 (0.35-4.56)
	Intermed.	103 (97.2)	3 (2.8)	0.40 (0.08-1.88)	0.37 (0.07-1.81)	37 (34.9)	69 (65.1)	1.19 (0.61-2.30)	1.60 (0.77-3.35)	3 (2.8)	103 (97.2)	3.89 (0.98-18.99)	3.87 (0.92-19.84)
	High	80 (94.1)	5 (5.9)	0.86 (0.22-3.61)	0.71 (0.17-3.14)	26 (30.6)	59 (69.4)	1.45 (0.72-2.92)	1.69 (0.77-3.74)	2 (2.4)	83 (97.6)	4.70 (1.04-32.89)	6.35 (1.25-48.46)
**Literacy score**	Mean (SD)	10.3 (2.0)	9.9 (3.0)	0.92 (0.75-1.15)	0.91 (0.74-1.14)	10.4 (2.5)	10.2 (1.8)	0.96 (0.86-1.08)	0.94 (0.83-1.07)	9.7 (2.8)	10.3 (2.0)	1.13 (0.91-1.39, p=0.245)	1.15 (0.90-1.44, p=0.238)

5. Adaptation found during observations

Adaptation from the initial implementation plan was analyzed according to actor: midwives, laboratory staff or patient. The results present the adaptations common to both centers and specific adaptations that arose.

Table S10 – Principal adaptation after implementation

	**Overall**	**Center A**	**Center B**
**Adaptations made by midwives**	No single-visit approach, 2-visit approach used to fit the internal organization<br>Screening activity aligned with laboratory activity	After 12:00 pm, women were asked to come back the next day	Results were systematically given the next day
**Adaptations made by laboratory staff**	Time set to close sampling reception	Sampling reception generally closed at 11:00 am<br>Laboratory staff managed to perform up to 12 test/day	Sampling reception closed at 10:00 am
**Adaptations made by patients**	Self-selection if arrived after a certain time		
